# *In vitro* comparative studies of resveratrol and triacetylresveratrol on cell proliferation, apoptosis, and STAT3 and NFκB signaling in pancreatic cancer cells

**DOI:** 10.1038/srep31672

**Published:** 2016-08-19

**Authors:** JingJing Duan, Wen Yue, JianYu E, Jyoti Malhotra, Shou-en Lu, Jun Gu, Feng Xu, Xiang-Lin Tan

**Affiliations:** 1 Rutgers Cancer Institute of New Jersey, Rutgers, The State University of New Jersey, New Brunswick, NJ 08904, USA; 2 Department of Pharmacy, 6th People’s Hospital South Campus, Shanghai Jiao Tong University, Shanghai 201499, P. R. China; 3 Department of Epidemiology, School of Public Health, Rutgers, The State University of New Jersey, Piscataway, NJ 08854, USA; 4 Department of Biostatistics, School of Public Health, Rutgers, The State University of New Jersey, Piscataway, NJ 08854, USA; 5 Wadsworth Center, New York State Department of Health, and School of Public Health, State University of New York, Albany, NY 12201, USA

## Abstract

Resveratrol (RES) has been studied extensively as an anticancer agent. However, the anticancer effects of triacetylresveratrol (TRES, an acetylated analog of RES) which has higher bioavailability have not been well established. We comparatively evaluated their effects on cell proliferation, apoptosis and the molecular changes in STAT3, NFκB and apoptotic signaling pathways in pancreatic cancer cells. Apoptosis was determined by flow cytometry. The nuclear translocation and interaction of STAT3 and NFκB were detected by Western blotting and immunoprecipitation, respectively. Both TRES and RES inhibited cell viability, and induced apoptosis of pancreatic cancer cells in a concentration and incubation time-dependent manner. TRES, similarly to RES, inhibited the phosphorylation of STAT3 and NFκB, down-regulated Mcl-1, and up-regulated Bim and Puma in pancreatic cancer cells. Remarkably, we, for the first time, observed that both TRES and RES suppressed the nuclear translocation, and interrupted the interaction of STAT3 and NFκB in PANC-1 cells. Comparative anticancer effects of TRES and RES on pancreatic cancer suggested that TRES with higher bioavailability may be a potential agent for pancreatic cancer prevention and treatment. Further *in vivo* experiments and functional studies are warranted to investigate whether TRES exhibits better beneficial effects than RES in mice and humans.

Pancreatic cancer, the fourth most common cause of cancer deaths worldwide, is one of the most enigmatic and aggressive human malignancies^1^. To date, surgical resection is the only potentially curative therapeutic option. However, due to absence of early symptoms and effective screening tests, the vast majority of pancreatic cancer patients has metastatic diseases at the time of diagnosis and therefore is not candidates for curative surgery^2^,^3^. Survival outcomes for patients with pancreatic cancer remain unsatisfactory with no significant improvement in pancreatic cancer incidence over the last decades. Thus, new approaches need to focus not only on improving outcomes for unresectable metastatic tumors, but also in prevention of pancreatic cancer^4^,^5^,^6^.

Resveratrol (RES, *trans*-3,5,4′-trihydroxystilbene, Fig. 1), a well-known natural polyphenolic phytoalexin present in grape skin and red wine, is a component of an Asian traditional medicine used to treat cardiovascular diseases^7^. RES has gained considerable attention as an anticancer agent since 1997^8^, because its potential use in chemoprevention and chemotherapy of various cancer forms, mediated via effects on cell growth, apoptosis, angiogenesis and metastasis^9^. Recently, numerous studies have shown its anticancer effects on pancreatic carcinoma^10^,^11^,^12^,^13^. To discover potential chemopreventive agents with increased bioavailability, more powerful or more selective biological effects, several polymethoxy and polyhydroxy derivatives of RES have been reported as anticancer agents against various human cancer cells^14^,^15^,^16^,^17^,^18^,^19^,^20^. In our previous study, we discovered a RES analog, 3,5,4′-tri-*O*-acetyl-trihydroxystilbene (Triacetylresveratrol, TRES, Fig. 1), as a potential chemoprevention agent by a gene expression-based, high-throughput screening of an 800-compound plant-derived library using induction of phase II enzymes in normal human lung cells^21^. However, few studies have been conducted to investigate the anticancer effects of TRES.

The development of pancreatic cancer is very complex, and has been suggested to be attributed to persistent low-grade inflammation. Two major inflammatory mediators, signal transducer and activator of transcription 3 (STAT3) and nuclear factor kappa B (NFкB), play a critical role in pancreatic carcinogenesis. Constitutive activation of STAT3 has been reported in 30% to 100% of human pancreatic ductal adenocarcinoma (PDAC) specimens, as well as in many PDAC cell lines^22^,^23^. Moreover, loss of STAT3 reduces acinar-to-ductal metaplasia and pancreatic intraepithelial neoplasia formation induced by oncogenic Kras^24^,^25^. NFκB is constitutively activated in 70% of human pancreatic cancer and in many human pancreatic cancer cell lines, but not in normal pancreatic tissues or in immortalized, nontumorigenic pancreatic epithelial cells^26^. Inhibition of constitutive NFκB activity by a phosphorylation defective IκBα (S32, 36A; IκBαM) suppresses pancreatic tumorigenesis in an orthotopic nude mouse model^27^. Interestingly, cross-talk between STAT3 and NFκB pathways has been suggested through the release of IL-6 and other cytokines and the autocrine/paracrine activation of cytokine receptors. In fact, NFκB and STAT3 co-regulate numerous oncogenic and inflammatory genes, such as Myc, Bcl-xL, cyclin D1, COX-2, and IL-1β^28^. The dysregulation of these genes due to the persistent activation of both NFκB and STAT3 in tumors and tumor microenvironment is crucial for the progression of pancreatic tumor. These studies suggest that the NFκB/STAT3 signaling pathway plays a critical role in inflammation mediated pancreatic carcinogenesis.

In addition, cancer cells evolve diverse strategies to evade apoptosis by disturbing the intrinsic apoptotic pathway. Apoptotic cell death is tightly regulated by Bcl-2 family protein members, including anti-apoptotic proteins, such as Bcl-2 and Mcl-1 and pro-apoptotic proteins such as Bim and Puma. Cancer cells can achieve the goal of evading apoptosis by increasing the expression level of anti-apoptotic proteins, and/or down-regulating pro-apoptotic proteins^29^. Moreover, several anti-apoptotic proteins, such as Bcl-2 and Mcl-1, which are known to be crucial for cancer cell survival, are direct target genes of STAT3 and NFкB. The effects of RES on inducing apoptosis in pancreatic cancer cells by multiple molecular targets in STAT3 or NFкB inflammatory pathways have been reported^12^,^30^, however, the effects of TRES on inducing apoptosis, and the effects of RES and TRES on cooperation and interaction between STAT3 and NFкB, which may contribute to inducing apoptosis in pancreatic cancer cells, have not been investigated.

In this study, we hypothesized that TRES may be effective in inducing apoptosis and inhibiting NFкB-STAT3 signaling pathways in pancreatic cancer cell lines. We first quantitatively detected the effects of TRES, in comparison to RES, on inhibiting cell viability and inducing apoptosis in the human pancreatic cancer cell lines, PANC-1 and BxPC-3. Furthermore, we evaluated the effects of RES and TRES on the modulation of the key molecular targets in STAT3 and NFкB, as well as their interaction and translocation into the nucleus of pancreatic cancer cells.

## Results

### Effects of resveratrol and triacetylresveratrol on cell viability of PANC-1 and BxPC-3 cells

**Figure 2:**
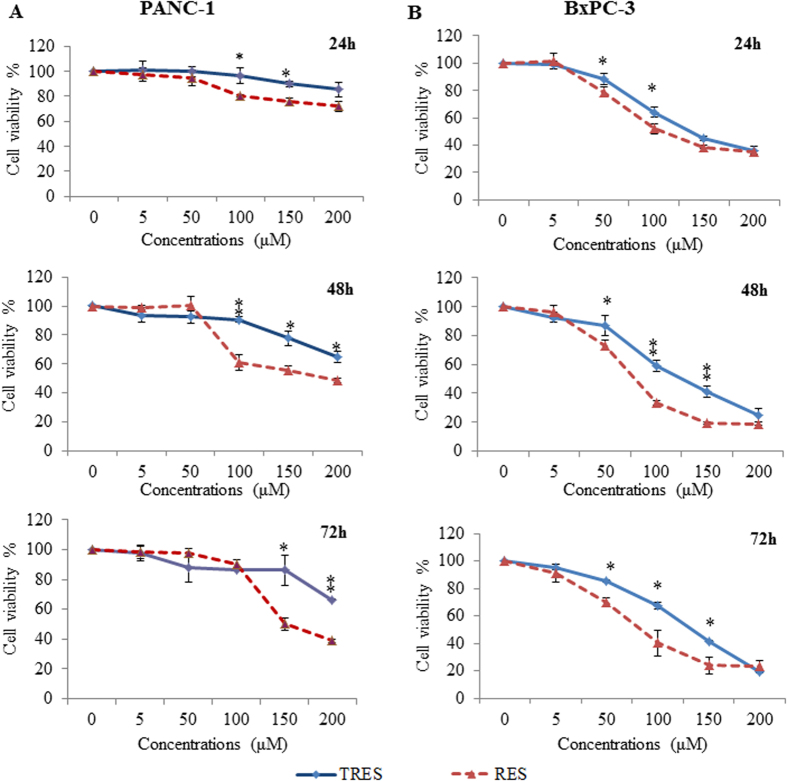
Triacetylresveratrol and resveratrol inhibited cell viability in a dose- and time- dependent manner. PANC-1 and BxPC-3 cells were treated with triacetyresveratrol or resveratrol at indicated concentrations for 24, 48, and 72 h. Cell viability was evaluated by MTS assay. Date are means ± SD, n = 3 means of triplicate measures. ^*^P < 0.05, ^**^P < 0.01, compared to RES. TRES, triacetylresveratrol; RES, resveratrol.

To evaluate the effects of RES and TRES on cell viability, we performed MTS assay by exposure of PANC-1 and BxPC-3 cells to TRES and RES at a concentration range of 0–200 μM for 24 h, 48 h and 72 h. We observed that both TRES and RES significantly suppressed cell growth in dose- and time- dependent manner in both cells (Fig. 2). BxPC-3 cells were more sensitive to TRES and RES, especially, when the concentrations of them reached 50 μM after 48 h. Regarding the agent effects, RES had significantly stronger effects on inhibition of cell viability in both PANC-1 and BxPC-3 cells across the three time points, compared to TRES. For example, after treatment with 100 μM TRES and RES for 48 h, the cell viability of PANC-1 cells were 90.66 ± 1.89% and 60.81 ± 5.39% (P < 0.01), and the cell viability of BxPC-3 cells were 56.94 ± 2.10% and 34.11 ± 1.38%, respectively (P < 0.01).

### Comparative effects of resveratrol and triacetylresveratrol on apoptosis

**Figure 3:**
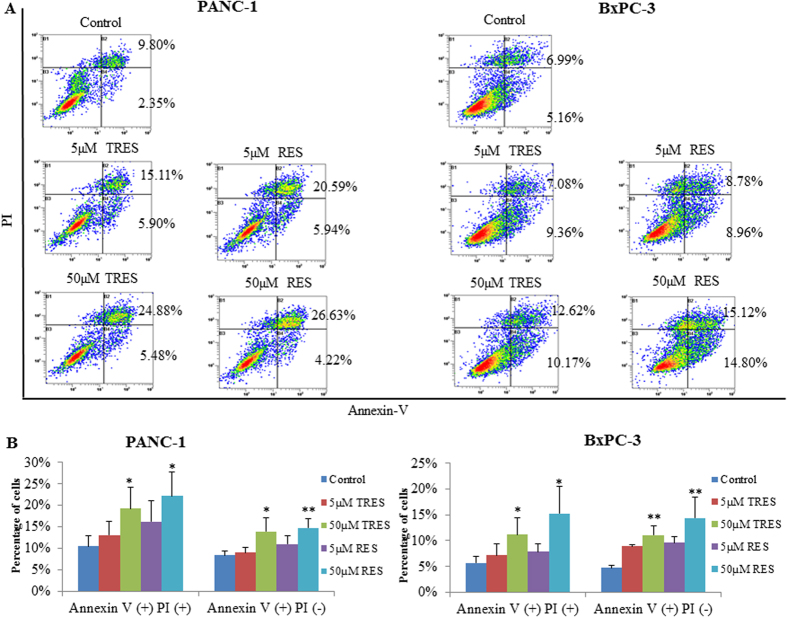
Triacetylresveratrol and resveratrol induced apoptosis determined by analysis of apoptotic cells using flow cytometry. PANC-1 and BxPC-3 cells were stained with Annexin-V-FITC and PI following treatment with or without triacetylresveratrol or resveratrol at indicated concentrations for 48 h. Apoptosis was determined by flow cytometry. (**A**) The results were analyzed by flow cytometry; (**B**) The results were from statistical analysis of three independent experiments. ^*^P < 0.05, ^**^P < 0.01, compared to the untreated control. TRES, triacetylresveratrol; RES, resveratrol.

To define the type of cell death induced by TRES, we assessed the apoptotic cell death in both PANC-1 and BxPC-3 cells by flow cytometry using co-staining of Annexin-V and propidium iodide (PI). Both PANC-1 and BxPC-3 cells were treated with different concentrations of TRES or RES (5 and 50 μM) for 48 h. The results demonstrated that both TRES and RES could induce apoptosis in dose-dependent manner in PANC-1 and BxPC-3 cells (Fig. 3A). Compared to the untreated control, TRES and RES at the concentration of 50 μM could significantly induce apoptosis in PANC-1 and BxPC-3 cells, and resulted in about 56% and 82% increases in apoptotic PANC-1 cells, and about 88% and 146% increases in apoptotic BxPC-3 cells, respectively (Fig. 3B). No significant difference between TRES and RES on the induction of apoptosis was observed in our studied conditions.

**Figure 4:**
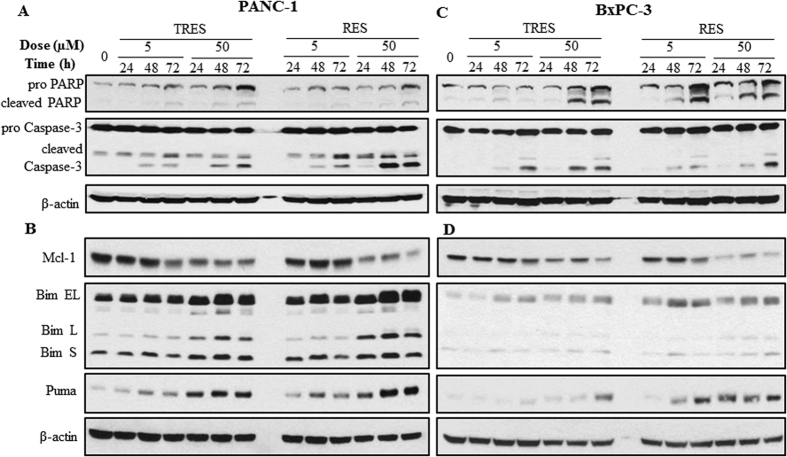
Effects of triacetylresveratrol and resveratrol on apoptotic signaling pathways. PANC-1 and BxPC-3 cells were treated with 5 or 50 μM triacetylresveratrol, or resveratrol for 24, 48, and 72 h. (**A**) The expression of PARP, Caspase-3 and their cleavage were detected by western blotting. (**B**) The expression of anti-apoptosis and pro-apoptosis proteins in PANC-1 and BxPC-3 cells were determined by western blotting using indicated antibodies, and β-actin was used as a loading control. TRES, triacetylresveratrol; RES, resveratrol.

Furthermore, we determined the apoptosis by measurement of the cleavage of PARP and caspase-3, which are the hallmarks of apoptosis. Both TRES and RES, at the higher dose (50 μM), significantly induced the cleavage of PARP and caspase-3 in PANC-1 and BxPC-3 cells (Fig. 4A,C). To further explore the mechanism of the apoptosis induced by RES and TRES, we investigated the protein levels of Bcl-2 family members. We first examined the anti-apoptotic protein Bcl-2 and Mcl-1 in PANC-1 and BxPC-3 cells. The protein levels of Mcl-1 decreased in the cells treated with either TRES or RES (Fig. 4B,D); however, no significant changes on the protein level of Bcl-2 were observed in both cells (data not shown). We also determined the protein levels of pro-apoptotic Bcl-2 family members, such as Bim and Puma, and both of them showed significant increases in the cells treated with TRES or RES (Fig. 4B,D). These results suggested that the down-regulation of Mcl-1, and the up-regulation of Bim and Puma could be involved in the apoptosis induced by RES and TRES in pancreatic cancer cells.

### Effects of resveratrol and triacetylresveratrol on STAT3 and NFκB signaling pathways

**Figure 5:**
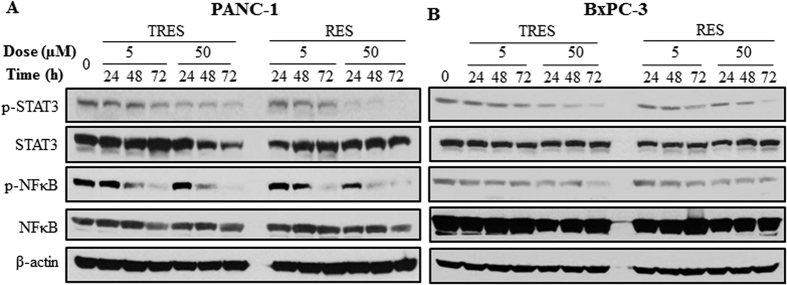
Effects of triacetylresveratrol and resveratrol on modulation of STAT3 and NFκB. PANC-1 and BxPC-3 cells were treated with 5 or 50 μM triacetylresveratrol, or resveratrol for 24, 48, and 72 h. Antibodies that detect the phosphorylated state of STAT3 at Tyr705 and NFκB at Ser536 were used, and the expression of their proteins were determined by western blotting using indicated antibodies, and β-actin was used as a loading control. TRES, triacetylresveratrol; RES, resveratrol.

Bcl family (Bcl-2, Mcl-1), which are known to be crucial for cancer cell survival, are prominent targets for STAT3 and NFκB, and down-regulated as a consequence of STAT3 and NFκB inhibition^31^. Therefore, we further investigated whether RES and TRES inhibited cell proliferation and induced apoptosis in pancreatic cancer cells by suppressing STAT3 and NFκB signaling pathways. We found that treatment with RES and TRES decreased the phosphorylation of STAT3 and NFκB in a dose- and time- dependent manner in PANC-1 and BxPC-3 cells, although no significant changes on the total STAT3 and NFκB were observed (Fig. 5). These results suggested that TRES and RES induced cell growth inhibition and apoptosis of cancer cells at least in part by inhibiting STAT3 and NFκB signaling in PANC-1 and BxPC-3 cells.

### Resveratrol and triacetylresveratrol suppressed the translocation of STAT3 and NFκB

**Figure 6:**
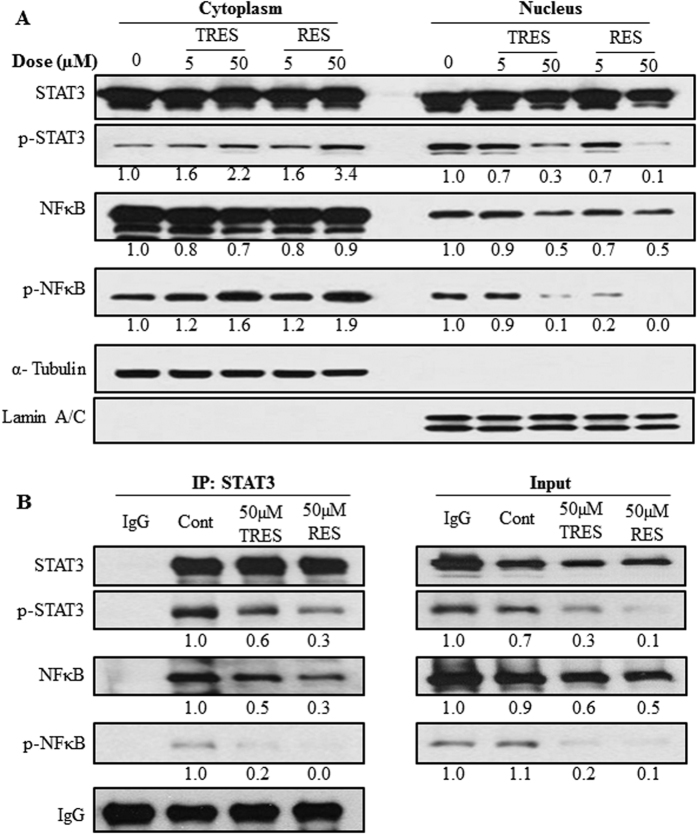
Effects of triacetylresveratrol and resveratrol on translocation and interaction of STAT3 and NFκB in PANC-1 cells. (**A**) PANC-1 cells were treated with 5 or 50 μM triacetylresveratrol, or resveratrol for 72 h. Cytoplasmic and nuclear fractions of treated cells were extracted and analyzed by western blotting to determine STAT3 and NFκB levels. (**B**) PANC-1 cells were treated with 50 μM TRES or RES for 72 h. STAT3 was immunoprecipitated from the whole cell lysates and analyzed for the presence of NFκB. TRES, triacetylresveratrol; RES, resveratrol.

Phosphorylation of STAT3 and NFκB induces their dimerization and translocation from the cytoplasm into the nucleus^32^,^33^. Therefore, we further determined whether RES and TRES affect the nuclear translocation of STAT3 and NFκB. For this purpose, PANC-1 cells were untreated or treated with TRES (5 or 50 μM) or RES (5 or 50 μM) for 72 h, and then we extracted cytoplasmic and nuclear fractions of the treated and untreated cells subjected them to Western blotting analysis. We found that both TRES and RES increased the protein levels of the phosphorylated STAT3 and NFκB in the cytoplasm, and decreased the protein levels of them in the nucleus (Fig. 6A). In addition, TRES and RES significantly decreased the protein levels of NFκB in the nucleus, but no changes on STAT3.

### Resveratrol and triacetylresveratrol interrupted the interaction between STAT3 and NFκB

Although we found that both RES and TRES could affect STAT3 and NFκB signaling pathways by inhibiting the phosphorylation and nuclear translocation of STAT3 and NFκB, the connection between these two pathways were still unknown. Several NFκB family members, in particular RelA/p65 and p50, were found to physically interact with STAT3, and this interaction may result in either specific transcriptional synergy or repression of NFκB/STAT3 regulated genes^34^. Therefore, we conducted immunoprecipitation to examine the effects of RES and TRES on the interaction of STAT3 with NFκB in PANC-1 cells. We immunoprecipitated endogenous STAT3 protein from the cells and determined its binding to NFκB and the phosphorylated NFκB using Western blotting. We observed that total NFκB and the phosphorylated NFκB binding to STAT3 significantly decreased after the treatment with 50 μM TRES or RES for 72 h (Fig. 6B). These results suggested that TRES and RES could also interrupt the interaction between STAT3 and NFκB, indicating that RES and TRES may be function as inhibitors of NFκB:STAT3 interaction.

## Discussion

In this study, both RES and TRES inhibited cell viability in a dose- and time-dependent manner, and TRES had comparative effects of RES on inducing apoptosis by the down-regulation of Mcl-1, and the up-regulation of Bim and Puma, in pancreatic cancer cells. We also found that both TRES and RES inhibited the phosphorylation of STAT3 and NFκB and suppressed the nuclear translocation of the phosphorylated STAT3 and NFкB. Remarkably, we, for the first time, showed that RES and TRES interrupted the interaction of STAT3 and NFκB in PANC-1 cells.

We observed that RES and TRES significantly down-regulated anti-apoptotic Bcl-2 family protein Mcl-1 and up-regulated pro-apoptotic Bcl-2 family proteins Bim and Puma. Mcl-1 amplification is one of the most common genetic aberrations observed in human cancers, including pancreatic cancer^35^,^36^, indicating Mcl-1 plays a key role in pancreatic tumorigenesis. We also detected the expression of another anti-apoptotic Bcl-2 family member and Bcl-2, however, RES and TRES didn’t down-regulate their expression in pancreatic cancer cells (data not shown). Bhardwaj *et al*. showed that RES down-regulated the expression of Bcl-XL and Bcl-2 in myeloma cells^37^. This discrepancy might be explained by the cell-specific effects of RES and TRES on Bcl family. In addition, RES and TRES up-regulated pro-apoptotic Bcl-2 family proteins Bim and Puma. Roy *et al*. showed that RES up-regulated the expression of Bim in PANC-1 cells^38^, which is consistent with our findings; however, no data has previously indicated that RES can up-regulated the expression of Puma in pancreatic cancer cells.

STAT3 is a key player in inflammation-related tumorigenesis, including pancreatic cancer, by promoting tumor cell proliferation, survival, invasion, angiogenesis, and metastasis^28^,^39^. NFκB has an important effect on cell growth and inhibition of apoptosis. Activation of NFκB also promotes proliferation, inflammation, tumorigenesis in cancer^40^. Aberrant STAT3 or NFκB signaling in malignant cells thus represents a promising therapeutic target. Our findings showed that RES significantly inhibited the phosphorylation of STAT3 and NFκB in pancreatic cancer cells. In consistent with our findings, Kotha *et al*.^30^ and Li *et al*.^12^ reported that RES inhibited the phosphorylation of STAT3 in PANC-1 cells, and inhibited the phosphorylation of NFκB in PANC-1 and BxPC-3 cells, respectively. We also observed the comparative effects of TRES on inhibiting the phosphorylation of STAT3 and NFκB, suggesting that TRES could also be an effective inhibitor of STAT3 and NFκB.

STAT and NFκB are activated by different pathways and migrate to the nucleus to bring about a transcriptional activity. Constitutively activated STAT3 and NFκB by phosphorylation or acetylation in tumor cells are associated with the development and progression of cancers, after translocation into the nucleus^41^,^42^. Kim *et al*. showed that RES inhibited the nuclear translocation of STAT3 and STAT5 in renal cell carcinoma^43^, and Wen *et al*. reported that inhibition of NFκB nuclear translocation committed resveratrol-treated medulloblastoma cells to apoptosis^44^. However, there is no data showing whether RES affects the nuclear translocation of STAT3 and NFκB in pancreatic cancer cells. In order to investigate whether RES and TRES induce apoptosis of pancreatic cancer cells by STAT3 or NFκB signaling pathway, we made cytoplasmic and nuclear fractions of PANC-1 cells treated with TRES or RES and analyzed them by Western blotting to measure the levels of STAT3 and NFκB signaling molecules. We found that RES and TRES increased the levels of the phosphorylation of STAT3 and NFκB in the cytoplasm and decreased those in the nucleus. Interestingly, they also reduced the level of NFκB, but not STAT3, in the nucleus. These results suggested that TRES and RES suppressed the translocation of the phosphorylated STAT3 and NFκB into the nucleus, thus down-regulated the downstream target genes of STAT3 and NFκB, such as Mcl-1.

We observed that the interaction between STAT3 and NFκB in pancreatic cancer cells were interrupted by the treatment with 50 μM TRES or RES for 72 h in PANC-1 cells. Indeed, the activation and physical interaction between STAT3 and NFκB were found in many human squamous carcinoma and cancer cells^34^,^45^,^46^. STAT3 and NFκB act as two major transcriptional factors to link inflammation with tumorigenesis, both of them are rapidly activated in response to various stimuli including stresses and cytokines. After being activated, they control the expression of anti-apoptotic, pro-proliferative and immune response genes. Some of these genes overlap and require transcriptional cooperation between the two factors^34^. STAT3 and NFκB thus bind to a subset of gene promoters to collaboratively induce their target genes expression. Through their functional interaction, STAT3 and NFκB collaboratively promote tumor development via induction of pro-tumorigenic genes^47^. To the best of our knowledge, this is the first study to show that RES and TRES interrupted the interaction between STAT3 and NFκB, indicating that TRES and RES may be function as inhibitors of NFκB:STAT3 interaction to suppress their malicious cooperation in pancreatic cancer cells. STAT3 can translocate into the nucleus only when it is phosphorylated. Since RES and TRES inhibited the phosphorylation step, STAT3 could not translocate into the nucleus, hence could not interact with NFκB there. In fact, we observed that RES and TRES increased the levels of the phosphorylated STAT3 and NFκB in the cytoplasm and decreased those in the nucleus. It is possible that the inhibition of STAT3-NFκB interaction by RES and TRE is due to the decreased interaction between STAT3 and NFκB in the nucleus. It is worthy investigating whether there is a differential interaction model between cytoplasmic and nucleus STAT-NFκB proteins upon RES and TRES treatment.

RES has been extensively studied, and has been shown to possess a cancer chemopreventive^8^ and chemotherapeutic potential^48^. Studies indicated that RES may induce growth arrest and apoptosis through a number of potential mechanisms including: inducing p53 activation mediated by ERKs- and p38 kinase^49^, targeting the Forkhead box O transcription factors through the inhibition of PI3K/AKT and MEK/ERK signaling^38^, and targeting the hedgehog pathways^13^. A recent study suggested RES induced apoptosis and cell cycle arrest via modulation of ERK1/2 and GSK3β pathways in human pancreatic cancer cell line AsPC1 without phosphorylated STAT3 and NFκB^50^. While these differences indicate a dependence on cell type, these findings are consistent with our *in vitro* studies which suggested that inhibition of cell proliferation may be an essential mechanism to prevent pancreatic carcinogenesis by RES.

However, the anti-inflammatory and anticancer effects of RES are limited by its low oral bioavailability^51^,^52^. It was suggested to modify its molecule in order to enhance its bioavailability while preserving its biological activity. A number of synthesized chemical analogs by modification in hydroxyl groups of RES or its hydroxyl groups’ positions, such as polymethoxy and polyhydroxy derivatives^16^,^18^,^19^,^20^, have been reported as anticancer agents with the same or even higher inhibitory effects against various human cancer cell lines. In this study, we found that a novel resveratrol analog, TRES, showed the anticancer activities similar to RES. It has been reported that TRES, compared to RES, has improved pharmacokinetic properties with longer half-life, increased AUC and volume of distribution^53^. Additionally, TRES was easier to transfer to and interact with phospholipid bilayers, probably due to its higher hydrophobic nature, compared to RES^54^. Further *in vivo* experiments and functional studies are warranted to investigate whether TRES exhibits better beneficial effects than RES in mice and humans.

## Materials and Methods

### Cell lines

Human pancreatic cancer cell lines, PANC-1 and BxPC-3 were purchased from the American Type Culture Collection (Manassas, VA, USA). PANC-1 cells were maintained in Dulbecco’s Modified Eagle’s Media (DMEM) (Sigma-Aldrich, St Louis, MO, USA) supplemented with 10% heat-inactivated fetal bovine serum (FBS) (Sigma-Aldrich, St Louis, MO, USA). BxPC-3 cells were maintained in RPMI-1640 medium (Sigma-Aldrich, St Louis, MO, USA) containing 10% FBS. Both cell lines were grown in an incubator in 5% CO_2_ at 37 °C.

### Antibodies and reagents

Antibodies against STAT3, phosphor-STAT3 (Tyr705), NFκB, phosphor-NFκB p65 (Ser536), Mcl-1, Bim, Puma, PARP, Caspase-3, β-actin were purchased from Cell Signaling Technology (Beverly, MA, USA). Antibodies against Bcl-2 and Protein A/G Agarose beads were purchased from Santa Cruz Biotechnology (Santa Cruz, CA, USA). Antibodies against α-Tubulin and Lamin A/C were purchased from Active Motif (Carlsbad, CA, USA). Resveratrol and triacetylresveratrol were purchased from Sigma-Aldrich (St Louis, MO, USA).

### Cell viability

Cells (5000 cells per well) were plated into 96-well plates for 24 h and then treated with indicated concentrations of RES or TRES for additional 24 h, 48 h or 72 h. Cell viability was assayed using CellTiter96 AQ nonradioactive Cell proliferation kit (Promega, Fitchburg, WI, USA) according to the manufacturer’s instructions. The percentages of surviving cells from each group were calculated relative to controls. Controls were defined as 100% survival.

### Apoptosis assay

Apoptosis was determined by using an Annexin V-FITC/ PI apoptosis detection kit (BD Biosciences) according to the manufacturers’ instructions. Briefly, cells were treated with the indicated dosage of RES or TRES for 48 h. The untreated and treated cells were washed with PBS buffer and gently suspended in Annexin V binding buffer and incubated with Annexin V-FITC (20 μg/mL) and PI (20 μg/mL) for 15 minutes in the dark. Flow cytometry analysis was performed using Cellquest software.

### Western blotting

The treated and untreated cells were rinsed twice with ice-cold PBS and extracted on ice with cell lysis buffer (Cell signaling, Beverly, MA, USA) which contains 20 mM Tris-Hcl (pH 7.5), 1% Triton, 150 mM NaCl, 1 mM EDTA, 1 mM EGTA, 1 mM Na3VO4, 2.5 mM sodium pyrophosphate, 1 mM beta-glycerophosphate, 1 μg/ml leupeptin and 1 mM PMSF. The protein concentrations of lysates are determined with BCA Protein Assay Kit (Thermo scientific, Waltham, MA, USA). A stock of the extract was made in 1 × Laemmli buffer (Bio-Rad, Hercules, CA, USA) and stored at −20 °C for western blot analysis. 20 μg of total proteins from each sample were loaded and separated on a gradient 8-12% polyacrylamide gel and transferred to polyvinylidene difluoride (PVDF) membrane. Membranes were blocked with 5% fat-free milk in Tris-buffered saline-Tween 20 (TBST, 20 mM Tris, pH 7.6, 137 mM NaCl, and 0.1% Tween 20) for 1 h at room temperature, followed by an overnight incubation at 4 °C with the primary antibodies. Blots were subsequently washed three times with TBST and then incubated with the appropriate HRP-conjugated secondary antibodies for 1 h at room temperature. After three additional TBST washes, the immunoreactive bands were visualized by enhanced chemiluminescence (Thermo Fisher, Rockford, USA) according to the manufacturer’s instructions.

### Cytoplasmic and nuclear fractionation

Cytoplasm and nucleus were isolated by using Nuclear Extract Kit (Active Motif, Carlsbad, CA, USA) according to the manufacturers’ instructions. Briefly, cells were treated with the indicated dosage of RES and TRES for 72 hours. The untreated and treated cells were washed with 5 mL ice-cold PBS, and collected by scraping and pelleted by centrifugation. Cells were transferred into a pre-chilled 15 mL conical tube, and gently resuspended in 500 μL hypotonic buffer by pipetting up and down several times. Cells were incubated on ice for 15 min, and the homogenates were centrifuged for 2 min at 14000× g at 4 °C. The supernatants (cytoplasmic fraction) were transferred and saved. Nuclear pellets were resuspended in 50 μL complete lysis buffer, and incubated on ice for 30 minutes. The homogenates were centrifuged for 10 min at 14000× g at 4 °C. The supernatants (nuclear fraction) were transferred and analyzed by Western blotting.

### Protein immunoprecipitation

After indicated treatments, cells were collected and lysed in cell lysis buffer with protease inhibitor cocktail on ice. 40 μL of protein A/G sepharose beads and 1 μg STAT3 or IgG antibody were added to the cell lysates containing 400 μg total proteins. Following overnight incubation with gentle rocking at 4 °C, the beads were washed with PBS buffer for 5 times and analyzed by Western blotting.

### Statistical analysis

The data from the cell viability assay were normalized to the untreated control (100% viability). We initially used three-way ANOVA to test the effects of agent, time, dose and their interaction. We then separately tested agent and dose response effect by one-way ANOVA with post hoc Dunnett’s test for comparing groups to the control in different time points. Results were expressed as means ± SEM, n = 3 means of triplicate measures. Similarly, two-way ANOVA was used initially to test the effects of agent, dose and their interaction on apoptosis and the modulations of protein expression, and one-way ANOVA with post hoc Dunnett’s test was used to test agent and dose response effect separately by comparing groups to the control. Results were expressed as means ± SEM, n = 2 means of triplicate measures.

## Additional Information

**How to cite this article**: Duan, J.J. *et al*. *In vitro* comparative studies of resveratrol and triacetylresveratrol on cell proliferation, apoptosis, and STAT3 and NFκB signaling in pancreatic cancer cells. *Sci. Rep*. **6**, 31672; doi: 10.1038/srep31672 (2016).

## Figures and Tables

**Figure 1:**
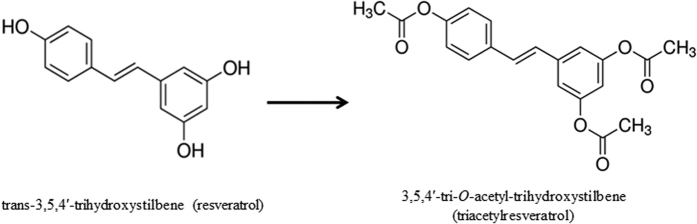
The chemical structure of resveratrol and triacetylresveratrol.
